# Parent-young people communication about sexual and reproductive health in E/Wollega zone, West Ethiopia: Implications for interventions

**DOI:** 10.1186/1742-4755-9-13

**Published:** 2012-08-16

**Authors:** Dessalegn W Tesso, Mesganaw A Fantahun, Fikre Enquselassie

**Affiliations:** 1 Department of Reproductive Health, Population and Nutrition, Addis Ababa University, P.O. Box 9086, Addis Ababa, Ethiopia; 2 School of Public Health, Addis Ababa University, P.O. Box 9086, Addis Ababa, Ethiopia; 3 Department of Epidemiology and Biostatistics, School of Public Health, Addis Ababa University, Addis Ababa, Ethiopia

**Keywords:** Parent, Young people, Communication, Culture, Taboo, Reproductive health

## Abstract

**Objectives:**

This study aims at examining parent-young people communication about sexual and reproductive health related topics and factors associated with it from both young people’s and parents’ perspectives.

**Methods:**

A cross-sectional study was conducted among 2,269 young people aged 10–24 years in Nekemte town and semi urban areas, western Ethiopia. Chi-square and multivariate logistic regression analyses were conducted using SPSS for windows version 16. The qualitative data was coded, and categorized in to emerging themes using the open code software version 3.4.

**Result:**

About a third of young people-32.5% (32.4% of females and 32.7% males) engaged in conversation about sexual and reproductive health topics with their parents/parent figures during the last six months. In logistic regression analyses, young people who were aged 15–19 years were more likely to report parent-communication compared to the other age groups (AOR = 1.57; 95%CI = 1.26-1.97). Female young people are more likely to discuss with their mothers, (AOR = 1.89, 95% CI = 1.13-3.2), sister (AOR = 2.16, 95% CI = 1.19-3.9) and female friends (AOR = 11.7, 95% CI = 7.36-18.7) while males are more likely to discuss with male friends (AOR = 17.3, 95%CI = 10-4-28.6). Educated young people were more likely to parent-communicate(AOR = 1.70, 95%CI = 1.30-2.24). Fear of parent, cultural taboos attached to sex, embarrassments, and parents’ lack of knowledge related to sexual and reproductive health were found to be barriers for parent communication. Parent-communication takes place not only infrequently but also in warning, & threatening way.

**Conclusion:**

Parent-young people communication about sexual health is occurring rarely in the family and bounded by certain barriers. Programmes/policies related to young people’s reproductive health should address not only individual or behavioral factors but also cultural and social factors that negatively influence parent-communication about reproductive health.

## Introduction

An increased incidence of HIV infection in adolescents has led researchers to examine factors that influence young people’s sexual behaviors. One of these factors is parent-adolescent communication about sexuality [1] Although sexual communication is a principal means of transmitting sexual values, beliefs, expectations, and knowledge between parents and children [2], discussions on sex-related matters are a taboo in Africa [3] and believed that informing adolescents about sex and teaching them how to protect themselves would make them sexually active [4].

In the same way, parent-youth communication on SRH issues, in Ethiopia, is believed to be culturally shameful [5]. Socio-cultural taboos attached to it and lack of proper knowledge makes open discussions about sexual and reproductive health topics difficult. This difficulty can be judged from study conducted, for example, in Zway, Ethiopia, that only 20% of parents reported to ever discussing sexual and SRH with their young people sometimes in the past [6]. However, it is believed that, home, as the initial focal point for investing in young people, is one of the many layers of environments for socialization. Providing avenues for child/parent connectedness, communication, and monitoring, the home is expected to serve as a stabilizing factor in the lives of young people [3,7].

Although, young people in Ethiopia constitute over one-third of the total population [8], most youth do not have access to information on issues that have great impact on their SRH [9,10]. The health seeking behavior of these people particularly in relation to their sexual and reproductive health in Ethiopia is very low [11]. In addition to these, the existing reproductive health (RH) services are adult-centered; thus making less accessible to these population [12]. Furthermore, health care providers in Ethiopia are often ill equipped to address adolescent-specific needs [13]. In such cases, the participation of parents, community members and other stakeholders is crucial to improve health status of the youth [14].

Nekemte town is characterized by high and ever increasing HIV/AIDS prevalence rate [15,16]. Thus, families, as primary socializing agent and live models for their children need to play an important role in shaping the sexual life of their offspring but only if parents were open, skilled and comfortable in having those discussion [17]. However, not much support is offered for parent communication, and parents often do not talk to their children because they feel confused, ill-informed, or embarrassed about these topics [18].

Although the government has identified RH of young people as one of the priority areas in The National RH Strategy taking the household and community as vehicles for change it is not yet put in practice [19]. The role of parent-young people communication about youth reproductive health and its current status is not well addressed while it is important to have a comprehensive community–based data on parent communication to help putting this strategy in to practice.

Thus, the purpose of this study was to: assess if parent communicate with their young people about sexual and reproductive health and circumstances under which this communication takes place with the associated barriers of communication.

## Methods

### Study area and population

The study was conducted in Nekemte and the surrounding three semi-urban kebeles in East Wollega Administrative zone, west Ethiopian, located at 331 km from Addis Ababa.

The source populations were never married in- and out-of-school young people aged 10–24 years with the inclusion criterion of never married and living in the area for at least six months at the time of the study. The study populations were all unmarried in-and-out-of-school male and female young people aged 10–24 and randomly selected to be included in the study. The participants of focus group discussions were purposely selected from in-and out-of-school young people, parents, school teachers and community leaders.

### Design and sampling procedures

A community-based cross-sectional house-to house and institution-based survey was conducted. The data was collected using a multistage systematic sampling method from the study area. The Kebeles (the smallest administrative unit in a sub city) were selected both from urban and semi-urban areas (the first strata), then each *kebele* was divided in to “*Gotts*” (the second strata). Household enumeration was carried out in all selected “*gott” (*the smallest sub-administrative unit in a kebele) in the selected *kebeles* prior to the data collection to identify the households with eligible young people. Each household was given identification number which was later used as sampling frame.

From urban area, four sub-cities, each having two *kebeles* and three *kebeles* from six semi-urban *kebeles* surrounding Nekemte town and within 10 km were randomly selected to be included in the study. These eleven kebeles then, divided in to several “*Gotts*” and representative “*Gotts*” were selected based on their population size of each kebeles. Then households were drawn from each “*Gott*” using systematic sampling until the desired numbers of households were included. Sample size was calculated for in-school an out-of- school separately using a single proportion formula. It was calculated with the assumption of 95%CI, 3% margin of error and 10% none response rate. Accordingly, 1500 of out–of–school and 845 in–school (7^th^-10^th^ grade) young people were required making the total sample size of 2345. The house numbers and class room role numbers were used as sampling frames. Male and females were sampled separately.

### Data collection

Data collection was conducted from February 1-May15, 2011. Data was collected using structured standard quantitative interview questionnaires adopted from Family Health [20]. The English version was translated into the regional language (Afan Oromo) then back to English by another person to ensure consistency of the instrument. Focus group discussions guide was prepared based on the objectives of the study. The quantitative interview was administered by 12 diploma graduate male and female data collectors recruited from the study area. The research team was recruited based on their level of education, previous experience in data collection, knowledge of local language and culture. Adequate training was given for six days by the researchers focusing on sampling, interview technique, ethical issues and safety of the participants and on maintaining confidentiality. The field data collection procedure was closely supervised by three trained supervisors (a health officer and two sociologists) and the principal investigator.

Qualitative research was used to complement the quantitative study to widen our insights about both parents’ and young people’s perspectives with regard to communication about sexual and reproductive health matters as such information couldn’t be collected through a quantitative study design [17]. Both male and female parents were include in the FGDs as we were interested to see the perceptions of both parents and young people from their own perspectives. Teachers and parents were included as they are the potential sex educators and socializing agents. Thirteen focus group sessions were conducted based on level of information saturation. Out of 13 FGD 6 were conducted among young people (3 with males and 3 with females), 4 were conducted with parents (2 with males and 2 with females) and 3 were conducted with male and female teachers. Male and female focus group discussions were facilitated by trained same gender moderators and note takers. Eight to twelve participants took part in each discussion lasting for 2–2:30 hrs.

The FGDs were conducted in private and quiet rooms in kebele offices where only the moderator, the note taker and the FGDs participants were present. The FGD used an open questions followed by possible probing questions. After some common introductory questions, the interviewers asked the participants’ opinions and perception about the young people’s sexual and reproductive health behaviors and parent-young people communication about reproductive health.

Ethical clearance was obtained from IRB of College of Health Sciences of Addis Ababa University and written permission was also obtained from the related institutions at each level before the study was conducted. Written consent (from survey participants) and verbal consent (from FGD participants) and/or assent were obtained from each participant. Instead of any personal identifiers, codes were used in questionnaires and focus group discussions to identify respondents. Advice was given for those who requested counseling on SRH to visit the near by health institutions.

### Measurements

The dependent variable was the composite score of parent- young people communication on 12 sexual and reproductive health related topics during the last six months. It was obtained by the question: “During the last six months, have you discussed on any of the following sexual and reproductive health related topics with your parents or parent figure?” Then the responses for each question were dichotomized as “yes” or “no”. We considered that the participants had discussed if they reported having discussed at least on one or more of the 12 listed topics with their parents in the last six months. Each of these topics was classified by the researchers in to one of the three themes [1] Biological aspect of sex comprised two topics(a) body change during puberty and (b) menstruation [2] Prevention aspects of sex comprised five topics (a)Abstinence (b)family planning (c) condom use (d) where to get condoms (e) relationship with the opposite sex (f) negotiating for safe sex [3] Risks associated with sexual behaviors comprised four topics (a) HIV/AIDS/STI (b) unplanned pregnancy (C) Abortion and (d) use of drugs/alcohol.

The following questions were used to guide instrument development and analysis: Do Parents communicate with their children/young people about sexual and reproductive health in the families? What are the common contents (topics) of this communication? Under what contexts (circumstances) this communication takes place? How frequently parents communicate with their children? At what age of the children parents usually start this communication? What are the common barriers to communication about sex and related topics? Is Parent-young people/children communication about these topics important? How do parents/young people feel about this communication?

### Statistical analysis

Of the total sample collected, 76(3.2%) were excluded from the analysis for incompleteness. The final sample for data analysis was 2,269; 1071 (47.2%) males and 1198 (52.8%) females; making the response rate 96.7%. The data were cleaned, coded and entered in to SPSS for window version 16. Chi-square analysis was used to test the relationship between categorical variables (sex, age, ethnicity, level of educational, living arrangement, parents’ marital status, and level of education) with topics discussed during parent communication about sex and reproductive health and proportions presented. Socio demographic characteristics were included in to regression model to control confounding. Significant variables (*α* < .05) at bivariate level were subsequently entered into multiple logistic regressions with 95%CI.

Each FGD had 6 to 12 participants and discussions lasted for an average of 2–2 ½ hours. The discussions were tape-recorded, transcribed verbatim in local language, Afaan Oromoo, and then translated into English. The texts were coded**,** categorized and sorted into emergent themes using open code software 3.4.

## Results

### Socio - demographic characteristics

The majority of the young people, 1,237 (54.5%), were in the age range of 15-19 years. The mean age was 18.59 (SD2.84) for males and 18.34 (SD 2.73) years for females (Table 1).

**Table 1:** Socio-demographic characteristics of in-and-out-of-school young people, Nekemte, West Ethiopia, 2012

**Variable**	**Male**	**Female**	**Total**
Sex (n= 2269)	1071 (47.2%)	1198 (52.8%)	2269(100%)
Age (n=2269) 10-14	86 (8%)	80(6.7%)	166 (7.3%)
15-19	558 (52.1%)	680 (56.7%)	1238 (54.6%)
20-24	422(39.9%)	429(36.6 %)	851 (37.5%)
Ethnicity (n=1424) - Oromo	1002 (93.6%)	1124 (93.8%)	2126 (93.7%)
Amhara	36 (3.4%)	40 (3. 3%)	76 (3.3%)
Gurageh	21 (2%)	24 (2%)	45(2%)
Others	12 (0.5%)	10 (0.8% )	22 (0.97% )
Religion denomination (n=2269) - Protestant	502 (46.2%)	614 (51.3%)	1116 (49.2%)
Orthodox	367 (34.5%)	406 (33.9 %)	773 (34.1%)
Islam	111 (10.4%)	99 (3.8%)	210 (9.3%)
Catholic	24 (2.2%)	34 (2.8%)	58 (2.6%)
Others	67(6.3%)	45(3.8%)	112(4.9%)
Living arrangement (n=2262)			
With both biological parents	611 (69%)	626 (52.3%)	1237 (54.7%)
With mother only	170 (19.1 %)	208 (17.4%)	378(16.7%)
With father only	27 (3.1%)	29 (2.4%)	55 (2.4%)
Alone	25 (2.3%)	67 (5.6%)	92 (4.1%)
With other relatives	238 (22.2%)	267 (22.3%)	505(22.2%)
Respondents level of education (n=2256)			
Primary (<5 )	47 (4.4%)	56(4.8%)	103(4.6%)
Junior (5-8)	251 (23.5%)	227 (19.3%)	478(21.2%)
High school (9-12)	601(56.3%)	643 (54.7%)	1244 (55.4%)
Tertiary	169 (15.7%)	250(21.3%)	419(18.6%)
Mother’s Education (n=2269) Not educated	456 (44.7%)	530 (45.6%)	986(45.2%)
1-4	163 (16%)	241(20.7%)	404 (18.5%)
5-8	200 (19%)	217 (18.7 %%)	417(19.1%)
9-12	159 (15. 6%)	146(12.6%)	305(14%)
Tertiary	42 (4.1%)	29(2.5%)	71(3.3%)
Fathers’ level of education No educated	239 (23.6%)	331 (28.5%)	570(26.2%)
(2266) 1-4	148 (14.6%)	182 (15.7%)	330(15.2%)
5-8	235 (23.2%)	256 (22.1%)	491 (22.6%)
9-12	299 (29.5%)	331(28.5%)	630 (29%)
Tertiary	91(9%)	60(52%)	151(7%
Parents’ Marital status(n=2263) Married	732(68.3 %)	792(66.1%)	1524(67.2%)
Separated	25(2.3%)	29(2.4%)	54(2.4)
Divorced	56(5.2%)	58(4.8%)	114(5%)
Widowed	258(24.1%)	319(26.6%)	577(25.5%)

Ethnically, the majority, 2126 (93.7%), were Oromos followed by Amhara, 76 (3.3%). By religion about half, 1116 (49.2%), were protestant Christian while about one-third, 773(34.1%), were Orthodox. The rest were catholic or other religion followers. One thousand two hundred thirty seven (54.5%), of the young people reported that they were currently living with both biological parents, while 378(16.7%) and 56 (2.5%) were living with mother and fathers respectively (Table 1).

One thousand two hundred forty four (55.1%) of the study population have educated to high school level while about one- fifth, 478 (21.2%) were at junior level. About equal proportion of males, 456 (44.7%) and of females, 530(45.6%), were from mothers having no formal education. More females (28.5%) were from non-educated fathers than males (23.6%). More than two-third, 1,524(67.3%), of parents of the study population were from married parents while one–fourth, 576(25.5%), were from divorced parents (Table 1).

### Parent-young people communication about sex and reproductive health

In the context of this paper, communication on sexual and reproductive health was defined as the young people who have talked about at least one sex and reproductive health-related topics with their parents or parent figures during the last six months[2]. The participants were given a list of 12 items related to sexual and reproductive health issues to respond (yes/no) whether these topics had ever come up when they talked with their parents/parent figures during their life time and the last six months. Eight hundred eighty two, (42.5%), of the participants reported to have ever had discussed on SRH matters with their parents/parent figures. Slightly more males (44.2%) than females (41%) reported to have ever had engaged in conversation with their parents/parent figures on topics related to reproductive health.

**Figure 1:**
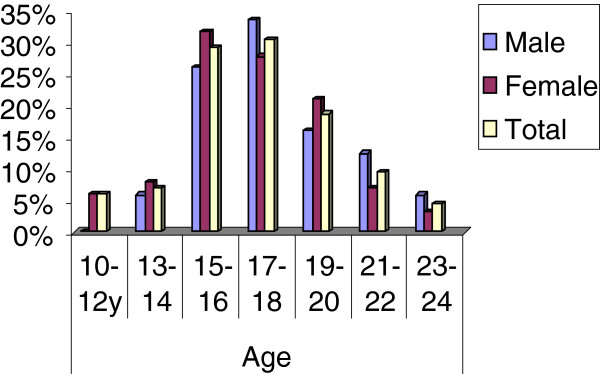
Parent communication about SRH by young people's age category, Nekemte, Ethiopia, 2012.

Seven hundred thirty eight (32.5%) or 32.4% of females and 32.7% of males reported to discuss with their parents on topics related to reproductive health during the past six months. However, differences have been observed across the age categories. Among younger people (10–14 years), only one-fifth, 18 (20.9%), of males and one–third of females, 27 (31.3%) reported parental communication. Males were less likely to discuss at early age than females of the same age group (P < 0.05). This proportion increases to one-third for both females (34.9% and males (37.1%) at age 15–19 years. Then, it tends to decline to 29.3% and 28.8% at age of 20–24 years for males and females respectively. Relatively more communication seems to occur at the age of 15-16 years for females and at 17–18 years males. (Figure 1).

Parent-young people communication on reproductive health related issues differs for both males and females with young people’s level of education. For males, it varies from 21.5%, for those young people educated to or less than 8^th^ grade to 37.3% for young people educated to high school and then shows a tendency to decline (36.7%) at tertiary level. It follows the same pattern for females which is 26.1%, 35.5%, 34% for the same education levels respectively.

Parent-young people communication about sexual and reproductive health was usually initiated by parents. This communication was positively associated with mothers’ and fathers level of education (Table 2). However, in logistic regression analyses, parent’s level of education showed no significant association with parents’ level of communication (Table 3).

**Table 2:** Socio-demographich characteristics and parent –young people communication about SRH during the last 6 months, Nekemte, west Ethiopia, 2012

	**Communicated with parents/parent figures in the last 6 months**			
**Variable**	**Male**		**Female**	
**New**	**Yes**	**No**	**Yes**	**No**
Sex	350(32.7%)	721(67.3%)	358(32.4%)	810(67.6%)
Age				
10-14	18(20.9%)	68(79.1%)	25(31.2%)	55(68.8%)
15-19	207(37.1%)	351(62.9%)	237(34.9%)	442(65.1%)
20-24	125(29.3%)	302(70.3%)	126(28.8%)	312(71.2%)
Respondents’ level of education				
1-8^th^ grade	64(21.5%)	234(78.5%)	74(26.1)	209(73.9%)
9-12^th^ grade	224(37.3%)	377(62.7%)	227(35.5%)	416(64.5%)
Tertiary	62(36.7%)	107(63.3%)	85(34%)	165(66%)
Residence area				
Urban	335(35.6%)	606(64.4%)	359(34.7%)	676(65.3%)
Semi-urban	10(9.2%)	99(90.8%)	19(14%)	117(86%)
Religion				
Catholic	7(29.2%)	17(70.8%)	15(44.1%)	19(55.9%)
Protestant	171(34.1%)	331(65.9%)	193(34.1%)	421(68.6%)
Muslim	31(27.9%)	80(72.1%)	33(33.3%)	66(66.7%)
Orthodox	113(30.8%)	254(69.2%)	130(32%)	276(68%)
Others*	28(41.8%)	39(58.2%)	17(37.8%)	28(62.2%)
Religion attendance				
Very often	120(46.3%)	275(34.1%)	259(32.7%)	534(67.3%)
Often	117 (45.2%)	420(51.9%)	119(32.7%)	245(67.3%)
Rarely	22(8.5%)	115(14.2%)	9(24.3%	28(75.7%)
Living arrangement				
Both parents	200(32.7%)	441(67.3%)	191(30.5%)	435(69.5%)
Mother alone	49(29%)	120(71.1%)	56(27.2%)	150(72.8%)
Father alone	10(37%)	17(63%)	7(25%)	21(75%)
Other relatives**	47(32.1%)	53(67.9%)	17(37.9%)	29(63.1%)
Father’s level of education				
No education	68(28.5%)	171(71.5%)	112(33.8%)	219(66.2%)
1-8^th^ grade	115(30%)	268(70%)	137(31.3%)	301(68.7%)
9-12^th^ grade+	155(39.7%)	235(60.3%)	133(34%)	258(66%)
Mothers’ level of education				
No education	128(28.1%)	328(71.9%)	165(31.1%)	365(68.9%)
1-8th grade	122(33.6%)	24(66.4%)	151(32.9%)	308(67.1%)
9-12^th^ grade+	69(41.1%)	99(58.9%)	57(38.3%)	92(61.7%)

**=Aunt, grand parents, uncle, sister, brother etc.

**Table 3:** Topics ever discussed by age category, Nekemte, West Ethiopia, 2012

**Topics discussed**	**Proportion distribution by respondents’ age**		
	**10-14**	**15-19**	**20-24**
1. Biological aspect			
● Body change during puberty	4(4.6%)	72(82.8%)	11(12.6%)
● Menstruation	----	25(71.4 %)	8(24.2%)
** vDiscussed at least on one topic**	**4(4.6%)**	**78(77.2%)**	**11(18.2%)**
2. Preventive aspects			
● Condom	2(8.3%)	16(66.7%)	6(25%)
● Where to get condom	-----	8(61.5%)	5(38.5%)
● Family planning	1(2.2%)	29(63%)	16(34.8%)
● Abstinence	3(2.6%)	99(84.6%)	15(12.8%)
● Relationship with the opposite			
● sex	2(4.7%)	28(65.1%)	13(30.2%)
● Negotiation for Safe sex	------	24(70.6%)	10(29.4%)
**v**Discussed at least on one topic	**8(9.5%)**	**188(66.5%)**	**68(24%)**
3. Consequence aspects /outcomes			
● Unwanted pregnancy	8(9.3%)	57(66.3%)	21(24.4%)
● Abortion	------	16(76.2%)	5(23.8%)
● HIV/AIDS	33(10.6%)	203(65.1%)	76(24.4%)
● Drugs/Alcohol	-----	8(66.7%)	4(33.3%)
**vDiscussed at least on one topic**	**231(60.6%)**	**150(39.4%)**	**287(67.8%)**

About one-third, 200 (32.7%) of males and females, 191(30.5%), living with both parents reported discussing on SRH topics with parent. Relatively a higher proportion of males living with father, (37%), and females living with other relatives, (37.9%), reported to discuss more SRH health topics than those young people living in other living arrangements (Table 2).

In this study, the frequency of attending religious ceremonies seems to promote parent-young people interaction. Among young people those who reported parent communication during the last six months, those who reported attending religious ceremony more frequently were more likely (59.4%) to report parent communication compared to those who reported infrequent attendance (35.7%). (Table 2).

### Topics discussed

A low proportion of both males, 57 (15%), and females, 44 (10.4%), reported to have discussed with their parents on biological aspect of sexual and reproductive health topics such as boy change during puberty (20.1% of males and 14.8% of females) while 5.7% of males and 10.4% of females reported discussing about menstruation. One hundred seventy eight (46.6%) of males and 190 (44.8%) of females reported to discuss on preventive aspects like: condom use (6.2% of males and 3.5% of females) and about family planning (8.2% of males and 10% of females). But about two-third of males, 231(60.6%), and females, 287(67.8%), reported to have discussed on associated risk aspects of sexual and reproductive health topics like unwanted pregnancy and HIV/AIDS (Table 4).

**Table 4:** People involved in communication about SRH with the young people by gender, Nekemte, West Ethiopia, 2012

**People involved in the communication**	**Proportion of people involved by respondents’ gender**			
	**Male**		**Female**	
	**Yes**	**No**	**Yes**	**No**
Mother	36(10.3%)	312(89.7%)	79(20.4%)	308(79.6%)
Father	32(9.2%)	316(90.8%)	22(5.7%)	366(94.3%)
Brother	38(10.3%)	310(89.7%)	26(6.7%)	361(93.3%)
Sister	22(6.3%)	326(93.7%)	61(15.7%)	327(84.3%)
Female friend	47(13.7%)	348(86.3%)	223(57.5%)	165(42.5%)
Male friend	199(57.2%)	149(42.8%)	28(7.2%)	360(92.8%)
Boy friend	-	-	60(17.5%)	320(82.5%)
Girl friend	58(16.7%)	289 (83.3%)	-	-
Teachers	32(9.2%)	316(90.8%)	32(8.2%)	356(91.8%)
Health workers	47(13.5%)	301(86.5%)	63(16.2%)	325(83.7%)
Other relatives	7(1.8%)	377(98.2%)	14(3.2%)	421(96.8%)

### People involved in the discussions about SRH

In this study, same sex discussion was observed. Female young people reported to discussed with mothers (20.4%) and sisters (15.7%) while male young people reported to have discussed with their fathers (10.3%) and sisters (10.3%). More communication takes place between mothers and daughters (20.9%) compared to fathers and sons (5.7%). Aunt, uncles and grand parents were the least family members (<5%) mentioned by young people as a source of information on SRH. Nevertheless, large proportion of the young people listed people out side of household members as a source of information about SRH, particularly their friends (59.5% for females and 55.1% for males) (Table 5).

**Table 5:** Odds of socio-demographic characteristics predicting parent-young people communication about sex & reproductive health topics in the last 6 months, Nekemte, West Ethiopia, 2012

**Variable**	**Discussed about SRH topics**		**OR95%CI**	
	**Yes**	**No**	**COR95%CI**	**AOR95%CI**
Respondents” Age				
10-14	43(5.8%)	123(8. %)	0.86(0.59-1.25)	1.32(0.81-2.14)
15-19	444(60.2%)	793(51.8%)	**1.37(1.14-1.65)**	1.57(1.26-1.97)**
20-24	251(34%)	61440%)	1.	1.
Residence				
Urban	694(96%)	1282(85.6%)	**4.03(2.71-6.0**	2.81(1.83-4.31)**
Semi-rural	29(4%)	216(14.4%)	1	1.
Respondents’ level of education				
1-8^th^ grade	138(18.7%)	443(28.9%)	1	1
9-12^Th^	451(61.1%)	793(51.8%)	**1.83(1.46-2.28)**	1.70(1.30-2.24)**
Tertiary	147(19.9%)	272(17.8%)	**1.74(1.32-2.29)**	1.84(1.30-2.60)**
Living arrangement				
With both parents	391(35%)	846(53%)	0.86(0.66-1.1)	0.96(0.54-1.56)
With mother	106(14.4%)	272(17.8%)	0.84(0.65-1.08)	0.99(0.75-1.31)
With father	18(2.4%)	38(2.5%)	0.97(0.54-1.74)	1,18(0.61-2.27)
With other relatives	189(25.6%)	316(20.7%)	**1.29(1.05-1.58)**	1.28(1.01-1.62)*
Living Alone	34(4.6%)	58(3.8%)	**1.0**	**1.0**
Attending religious services				
Every often	401(54.5%)	787(51.2%)	**1.0**	1.0
At least once a week	293(39.8%)	608(39.2%)	**1.36(1,11-1.7)**	1.38(0.92-2.1)
Rarely	42(5.7%)	132(8.6%)	**2.1(1.35-3.14)**	1.38(0.91-2.1)
Mother’s education				
No education	293(42.3%)	693(48.4%)	1	1.
1-8^th^ grade	273(39.5%)	549(38.3%)	1.18(0.96-1.44)	0.77(0.55-1.1)
High school^+^	126(18.2%)	191(13.3%)	**1.56(1.2-2.03)**	0.81(.06-1.1)
Father’s education				
No education	180(25%)	390(26.9%)	1	1.
1-8^th^ grade	252(35%)	569(39.1%)	0.96(0.76-1.21)	0.84(0.64-1.08)
High school^+^	288(43%)	493(34%)	**1.27(1.0-1.59)**	0.94(0.70-1.26)

** = P=0.001, * = P=0.05.

Young people gave different reasons for choosing the people whom they discussed with on SRH issues of which the following were found to be significant: (a) because they don’t punish like parents (P < 0.001), (b) are knowledgeable (P < 0.001), (c) they take time to listen (P < 0.001) and (d) have interest to discuss on SRH (P < 0.001). In Chi-square analyses, only limited ever discussed topics were found to be significant at alpha 0.05 like: HIV/AIDS (P < 0.014), abstinence (P < 0.04) unwanted pregnancy (P < 0.014) and body changes during puberty (P < 0.047).

### Perceived parents’ responsiveness to SRH related questions

Both male and female young people perceived that their parents are not positively responding to their questions related to sex and reproductive health. Among young females those who reported to communicate sexual and reproductive health issues with their mothers, 307(29.4%), only less than one-fifth (19%) perceived that their mothers would answer helpfully if they ask sexual and reproductive health related issues (P < 0.001). Nevertheless, 45.5% of female young people perceived that their mothers would turn away without giving them answer if they ask their mothers sex and RH related questions (P < 0.001). In the same way, about half, 49.4%, of the females perceived that their fathers would turn away without giving them answer if they ask the same questions (P < 0.001) (Table 5).

Similarly, among young males those who reported to communicate sexual and reproductive health issues with their mothers, 260 (28.4%), only 21(15%) perceived that their mothers would answer helpfully if they ask sexual and reproductive health related issues (P < 0.001). Half of the males (50.3%) perceived that if they ask their mothers sex and RH related questions, mothers would turn away with out giving them answer (P < 0.001) and 45.9% of the males perceived that their fathers would turn away with out giving them answer if they ask the same questions (P < 0.001).

### Communication barriers for sexual and reproductive health topics with parents

The reason for not discussing SRH issues with parents are shown in Table 6. These include: fear of parents, embarrassment to discussing with parents, taboo attached to sex and parents failure to give time to listen and parents lack interest to discuss. In Chi-square analyses, parents’ failure to give time to listen (P < 0.001) and parents’ lack of interest to discuss (<0.001) were found to be significant for females than for their male counterparts. More over, more that two-third (69.5%) of the young people perceived that discussing SRH matters with parents is difficult and these young people were less likely to discuses with their parents (P < 0.001).

**Table 6:** Odds of peoples involved in the discussions and reasons for not discussing, Nekemte, west Ethiopia, 2012

**People talked to young people**		**Communicated about SRH during the last six months**			
		**Yes**	**No**	**COR95%CI**	**AOR95%CI**
Mother	Male	36(10.3%)	312(89.7%)	1.0	1.0
	Female	79(20.4%)	308(79.6%)	2.23(1.47-3.38	1.89(1.13-3.2)*
Sister	Male	22(6.3%)	326(93.7%)	1.0	1.0
	Female	61(15.7%)	327(84.3%)	2.8(1.7-4.62)	2.16(1.19-3.89)*
Female friends-	Male	47(13.7%)	348(86.3%)	1.0	1.0
	Female	223(57.2%)	360(42.5%)	8.28(5.85-11.73)	11.7(7.36-18.7)**
Male friends	Male	199(57.2%)	149(48.8%)	11.8(11.2-25.4)	17.3(10.4-28.6)**
	Female	28(7.2%)	360(92.8%)	1.0	1.0
Reasons for not discussing SRH topics with parents					
Because I fear my parents	Male	231(55.5%)	185(44.5%)	1.0	1.0
	Female	75(19.6%)	307(80.4%)	**0.19(0.14-0.27**	077(0.40-1.5)
I feel embarrassed	Male	27(32.9%)	55(67.1%)	1.0	1.0
	Female	332(30.5%)	756(69.5%)	**0.89(0.55-0.98)**	0.62(0.35-1.1)
Discussing SRH issues with parent is taboo	Male	10(12.8%)	68(87.2%)	1.0	1.0
	Female	74(6.7%)	1024(93.3%)	**0.49(0.24-0.99)**	0.52(0.24-1.13)
My parents do not give me their time to listen	Male	12(15.4%)	66(84.6%)	1.0	1.0
	Female	94(8.5%)	1006(91.5%)	**0.51(0.26-0.98**)	0.44(0.20-0.96)*

**=P=0.001 *= P=0.05, 1.0= constant, AOR= adjusted odds Ratio, COR= Crude odds Ratio.

CI= Confidence interval.

Logistic regression analyses were also used to assess the association between people involved in the discussions and topics discussed. Young people who were educated to high school and tertiary level were more likely to communicate with their parents compared to those with lower level of education (AOR = 1.70, 95%CI = 1.30-2.24 Vs. AOR = 1.84, 95%CI = 1.30-2.60) respectively. However, young people who perceived that their parents do not give their time to listen were less likely to discuss with their parents (AOR = 0.44; 95%CI = 0.20-0.96). Regarding residential area, young people living in urban were more likely to report sexuality communication with parents than semi-urban dwellers (AOR = 2.81; 95%CI = 1.83-4.31) (Table 3).

Young people those who were aged 15–19 years were more likely to engage in communication with parents compared to the other age groups (AOR = 1.57; 95%CI = 1.26-1.97). Female young people are more likely to discuss with their mothers, (AOR = 1.89, 95% CI = 1.13-3.2), sister (AOR = 2.16, 95% CI = 1.19-3.9) and female friends (AOR = 11.7, 95% CI = 7.36-18.7) while males were more likely to discuss with male friends (AOR = 17.3, 95%CI = 10-4-28.6) (Table 6).

Evidences from the young people’s focus group discussions suggest that culture was one of the important challenges hindering parents’ communication about sexual and reproductive health matters. As the result, young people go to their peers to discuss on SRH issues to learn as they are easier and ready to discuss than with their parents. Participants believe that some parents do not know that they are responsible to teach their children about reproductive health and related issues, rather they expect it from others like school; but from practical point of view, schools are not doing that. As young people discussants pointed it out:

"Parents do not want to discuss reproductive issues with their children because most of the time such issues are culturally considered taboo; moreover, they think that discussing these things is the role of schools. But schools are not doing that. So youths go to their peers to discuss on such topics (male 21 yrs, OSY)."

"Parents do not discuss sexual and reproductive health issues with their young people. The problem is our social norm that defines it [sexual matters] as taboo (Female 21 yrs, OSY)."

There were some divergent ideas regarding parent adolescent-communication about reproductive health. Some discussants of the young people said that there is parent-adolescent communication, but the focus is narrow and lacks depth. Others said that RH is not an agenda for discussion in the family. According to the discussants, the level of parents’ knowledge was also questionable. These issues were pointed out as:

"Now days, some parents started to discuss and advise their children about HIV/AIDS. It is not like the past times in which parents were not talking about sexual issues (20 yrs, male, OSY)."

"Parents do not discuss. They may not know detail about reproductive health. They mostly (if any) discuss only about HIV/STI (Male 21 yrs, OSY)."

"No, I do not agree with this idea . There could be few parents, less than 25 percent, doing that. The majority of parents do not discuss about RH with their children (22 female OSY)."

"No parents take RH discussion as their regular agenda for discussion. They bring these issues to table only when they are influenced by certain circumstances. For example girls are facing problem during their first menstruation. This is a simple example for lack of communication (19 yrs male, OSY)."

Parents also supported the ideas raised by the young people discussants. According to the parent discussants, intergenerational, cultural and social norms and parental lack of knowledge on RH were the reasons for not discussing RH issues. However, the parents believed that the emergence of HIV/AIDS has positively influenced the occurrence of parent communication on RH. These were addressed by female parent discussants as:

"Most of the parents are not discussing reproductive health (RH) issues with youth because of lack of awareness on RH, cultural taboos attached to it, and lack of knowledge (35 yrs mother)."

"It is difficult to expect parents to discuss on RH issues with youth. This is the way we were brought up. Some young people consider their parents are ignorant (41 yrs mother)."

"Such discussion did not exist in the past times. But since the emergence of HIV/AIDS, parents have begun discussing on RH related issues with their family… Most parents openly discuss HIV related issues with their children (38 years Female Parent)."

One of the male parent discussants also stressed this issue as*:*

"In our culture, let alone to talk about sexual related issues with children, wife-husband communication on such issues is rare. This is one of the bad cultures we have. A wife even doesn't tell her husband that she is pregnant until it becomes physically visible. This tradition is passing from generations to generations in our society. Every body shies to openly talk about sexual matters (60 yrs, male parent)."

The other interesting result of the focus group discussions were the context or how parents say it and the circumstances under which this parent-young people communication takes place in the families*.* Parents have no regular schedule to discuss on sexual and reproductive health matters with their children. The way in which the communication takes place is also not in a friendly and persuasive two-way communication. Rather, it is a unidirectional and warning type of communication. These were stated in the focus group discussions as:

"Such discussions are taking place when something happens to young people in their locality. Like when pregnancy [premarital] and HIV related problems happens to a young people in the area, like abortion, and related complications and deaths occur to their neighbor's children, or heard it from Mass Medias. At the same time, the discussions are usually not friendly; rather it occurs in threatening and warning manner (48 yeas male parent)."

"As it is said, most families discuss with their children indirectly on sexual issues like: “you see? Ms X’s daughter has got pregnancy out of marriage or she gave birth out of marriage, she is a bad girl. Don’t be like her.”’ and so on (33 yrs, male parent)."

The range of the parent-young people communication seems narrow that is limited only to a few topics of RH like: HIV/AIDS and abstinence. It also seems gender biased focusing on females and on the importance of virginity and the norm.

"The most common topics of parent-young people discussion were: HIV, abstinence and pregnancy … because, the loss of virginity will cause problem in marriage. In the early days, girls who married with out being virgin were being sent back to their families on donkey’s back (as punishment). For fear of this practice, they (girls) respect their parents' advices to preserve their virginity. But this day, virginity has lost its importance. This has caused changes in the willingness of youth to discuss with their parents (59 yrs male parent)."

Both parents’ and young peoples’ focus group discussants agree with the importance of parental monitoring (whereabouts of children and young people during their free time and after school). Especially parents believed that its importance is not only for the families or young people, but for the nation at large. However, according to the FGDs participants, most parents are not monitoring their children. The parent discussants explained this situation as:

"“Parents' monitoring is very important. Unless parents monitor them (children), parents will not get an opportunity to communicate to.” (47 yrs male parent)."

"“Parental monitoring has a paramount importance, because in so doing, they can discuss important issues” (45 yrs female parent)."

"R6. Yes, the advantages of parents’ follow up are manifold. It helps the youth, their families and the country at large. (46 years male parent)."

## Discussion

This study assessed if parents communicate with their young people about sex and reproductive health, the depth, the circumstances, the frequency and the timing of the communication both from parents’ and young peoples’ perspectives. The people involved in the communication, topics discussed, barriers to communication and the responsiveness of the parents in communicating with young people about SRH related were also assessed

In this study, 882(42.5%) (44.2% of male & 41% of female) young people reported to have ever discussed on sexual and reproductive health topics with their parents or parent figures during their life time. This finding is much lower than the result of study done in Mexico [21] that 83.1% reported having spoken with their parents about sex relations However it is relatively larger than the finding of the study done in Zeway, Ethiopia that only 20% of parents reported to ever have discussed with their children [22]. This difference may be attributable to the difference in the study population that the study done in Zeway collected information from parents while the current study collected information from young people. Similar to previous study [23] males and females were equally likely to discuss about SRH during the last six months that about one-third of both females (32.4%) and males (32.7%) reported to have discussed with their parents on topics related to reproductive health. This finding is lower than from the study result done in Ghana that more (46%) of females than males (28%) often talked to family members about sexual matters [24].

A Study done in Tanzania showed that communication about sex was mainly with the same sex (mother- daughter and father-son [25]. Likewise, in the current study, young people preferred discussion with same sex on SRH matters. From family members, females are more likely to discuss with their mothers (20.4%) while male young people discussed more with their fathers and brothers (10.3%). Other extended family members like grand parents, uncles and aunts were the least (<5%) to be mentioned as the source of information on SRH. This is in agreement with other finding [24]. This could be attributed to the expansion of formal educations, that facilitates early union of young people with peers, and parents’ migration from their original residence areas seeking jobs and leaving grand parents behind, the role of traditional extended family as a socializing agent is being eroded.

In the current study, both males and females reported to discuss more with nonfamily members of the same sex friends. More than fifty percent of both males (58.7%) and females (57.3%) mentioned that they prefer to get sexual and reproductive health related information from their friends than from their parents. This is in agreement with other research results [26]. This may is because parents were not responsive to young peoples’ questions hence, young people opt their friends for information they need.

Although it is generally low, the level of communication relatively increases with respondents’ age. Earlier literature states that the extent of communication on sexual and reproductive health matters increase with age and continuing through young adulthood [27].

This study revealed that young people start sexual intercourse as early as 8–9 years of age. Again the large proportion of both males (73.4%) and females (80.2%) reported to start sexual intercourse between the ages of 15–19 years while parent communication starts late. For example, more than fifty percent of males (59.8%) and females (59.6%) reported to start discussions on SRH between the ages of 15–18. This may imply that parents increase the extent of communication when they suspect that their children might have started sex then communication starts to decline in the older young people as parents may assume that young people at this age are adults.

Nevertheless, this result should be taken with caution because at this age, either parents might have discussed on more topics intentionally based on their children’s age or parents might have increased communication as they were becoming aware that their children have started sex at this age. However, the over all results of the current study suggests that communication about sex was initiated earlier.

On the other hand, a large proportion (65.6%) of the young people reported that SRH related topics were rarely discussed in the family. They believed that the issue suddenly becomes a point of discussion only when related problems occur or seen among young people in the area; like when early pregnancy [premarital] and HIV related problems happens to a young people in the area, like abortion, and related complications and deaths occur to their neighbor's children, or heard it from Media.

This finding is also substantiated by the qualitative result that parent-young people communication about sex and RH is rare and begins late. Earlier studies also found that parent-adolescent communication about sex begins late and that communication was triggered by seeing or hearing something a parent perceived negative and would not like their child to experience it [20,22]. This supports the hypothesis that parent communication about sexual and reproductive health starts at late age when parents suspect that their children started love relationship which has a programmatic importance that parent should be educated to start communication at early age.

The range of parent-young people communication was narrow that only limited topics were coming up in the discussions. The most commonly reported topics of discussions were: HIV/STI, sexual abstinence, body change during puberty and unwanted pregnancy. Other SRH related topics like use of condom, negotiating for safe sex, menstruation and family planning were the least frequently coming up topics in the discussions. This finding is consistent with prior study [28] that parents mostly discuss on HIV/AIDS and abstinence. This may be because of the stigma attached to HIV/AIDS, while loss of virginity and premarital pregnancy is defaming the families (normative issue) as reflected in FGDs. On the other hands, it could be due to the fact that the issue of HIV is commonly presented on media or parents will tend to avoid talking about sex-related sensitive topics.

The influence of lack of perceived parental knowledge, intergenerational cultural taboos attached to sexual issues and comfort reinforce each other and made parent-young people communication challenging. The interesting finding of this study is that both parent and young people discussants perceived that the barriers to the communication arise both from parents and young people sides. According to literature [29], parents’ behavior can influence the young people’s behavior; however, as communication is bidirectional, parents’ behaviors could also be influenced by young people’s behaviors. Therefore, the contribution of both parents and the young people is important for the occurrence of quality communication.

As the study used different data collection methods and a variety of sources of data, this result gives a better and balanced picture of the situation. More over, this study used both the life time and the recent information (six months) to minimize recall bias.

This study has its own limitation in that the participants reply might have been affected by social desirability that may have affected the validity of the result. The fact that the design was cross sectional, may hinder the determination of causality of relationship in some instances.

## Conclusions

This study revealed that the proportion of young people who communicated with their parents was low and parent’s involvement in the communication was limited. Instead, the most important sources of information on SRH were none family members like friends. Both the quantitative and qualitative result showed that the range of the parent young people communication about SRH is narrow that only limited topics were being discussed. Most of reproductive health related topics were not being covered to enable young people develop basic knowledge to resist any advances. Parent communication occurs \infrequently and late. Embarrassment, fear of parents, non-responsiveness of parents and cultural taboos attached to SRH and non-acceptance of the young people were identified as the main barriers to open parental communication on sexual and reproductive health (SRH) matters.

This study has showed the level of parent-young people communication and contributing factors that will help policy/program managers in designing a tailored action to create supportive environment for parent young people communication about SRH.

### Recommendations

To enhance parents’ knowledge, sectors like health and education should provide continuous training on SRH matters to creating community dialogue and conversations regarding parent young people communication. Conducting sustainable advocacy works targeting parents and communities on young people’s sexual and reproductive health is also needed. Age appropriate educational health services are necessary for all young people to help them develop communication skills and responsible sexual behaviors. Teachers’ training on SRH is also needed to strengthen and facilitate school sex education.

Regarding predicting barriers of communication, an important research question can be raised from this study particularly from the qualitative part that” which perception matters, that of the parents, or the young people’s? A further exploration is needed.

## Competing interests

We declare that there are no financial or non-financial competing interests related to this study.

## Authors' contributions

All the three authors were responsible for designing, data processing, statistical analysis, interpretation and writing up the final article and gave the final approval of the manuscript to be published.
